# Models of β-amyloid induced Tau-pathology: the long and “folded” road to understand the mechanism

**DOI:** 10.1186/1750-1326-9-51

**Published:** 2014-11-18

**Authors:** Ilie-Cosmin Stancu, Bruno Vasconcelos, Dick Terwel, Ilse Dewachter

**Affiliations:** Catholic University of Louvain, Institute of Neuroscience, Alzheimer Dementia, Av. E. Mounier 53, Av. Hippocrate 54, B-1200 Brussels, Belgium; reMYND nv, Gaston Geenslaan 1, B-3001 Leuven, Belgium

**Keywords:** Amyloid, Tau, Alzheimer’s disease, Animal models, Amyloid cascade hypothesis, Synaptic dysfunction, Inflammation, Prion

## Abstract

The amyloid cascade hypothesis has been the prevailing hypothesis in Alzheimer’s Disease research, although the final and most wanted proof i.e. fully successful anti-amyloid clinical trials in patients, is still lacking. This may require a better in depth understanding of the cascade. Particularly, the exact toxic forms of Aβ and Tau, the molecular link between them and their respective contributions to the disease process need to be identified in detail. Although the lack of final proof has raised substantial criticism on the hypothesis per se, accumulating experimental evidence in in vitro models, in vivo models and from biomarkers analysis in patients supports the amyloid cascade and particularly Aβ-induced Tau-pathology, which is the focus of this review. We here discuss available models that recapitulate Aβ-induced Tau-pathology and review some potential underlying mechanisms. The availability and diversity of these models that mimic the amyloid cascade partially or more complete, provide tools to study remaining questions, which are crucial for development of therapeutic strategies for Alzheimer’s Disease.

**Electronic supplementary material:**

The online version of this article (doi:10.1186/1750-1326-9-51) contains supplementary material, which is available to authorized users.

## The amyloid cascade hypothesis and anti-Aβ directed therapies

Brains of Alzheimer’s Disease (AD) patients are characterized by the presence of amyloid plaques and neurofibrillary tangles (NFTs) as diagnostic hallmarks, in addition to reactive microgliosis and astrogliosis, synaptic and neuronal loss, and a marked brain atrophy [1–5]. Identification of mutations in amyloid precursor protein (APP) and presenilin (PS1/2) that are autosomal dominantly linked to early onset familial AD [6, 7], represented major milestones in AD research. The histopathological similarity between sporadic and early familial cases was taken as evidence for a common etiology of the disease. Because in vitro and in vivo data indicated that Early Onset Familial Alzheimer’s Disease (EOFAD) mutations give rise to the generation of more β-amyloid (Aβ) peptides or more amyloidogenic species, their accumulation was postulated to be the cause of the disease process. Hence, it was postulated that accumulation of more amyloidogenic peptides results in a cascade of events leading to increased inflammation, Tau pathology, synaptic and neuronal loss, ultimately responsible for the clinical symptoms of AD [1]. In contrast, exonic and intronic mutations in MAPT, the gene encoding Tau, are associated with neurodegenerative diseases, but not with AD [8, 9], and hence do not result in amyloid pathology.

Accumulating evidence for a causal role of amyloid peptides in AD etiology resulted in a quest for anti-amyloid directed targets, which was particularly pursued preclinically in APP and APP/PS1 overexpressing transgenic mice. These models develop robust amyloid pathology, gliosis and synaptic and behavioral deficits – including cognitive deficits –, parameters that were used as read-outs for successful preclinical trials. Identification of alpha-secretase (ADAM10 [10], TACE), beta-secretase (BACE1 [11]) and gamma-secretase (as a complex containing aph1, pen2, nicastrin, PS1 [12, 13]) and the analysis of their potential as therapeutic targets in preclinical models [14], prompted straightforward small molecule-based approaches. In addition, other non-secretase directed approaches were developed including the more unexpected, atypical approach of active and passive immunization against Aβ [15, 16].

## The amyloid cascade hypothesis: criticism raised by lack of successful clinical trials fueled interest in Tau-directed therapies

However, anti-Aβ directed clinical trials in AD patients have been incompletely satisfying or disappointing either by lack of amyloid lowering effects or by disappointing outcomes [17] including following immunization, i.e. marked removal of amyloid plaques without halting/slowing disease progression. Hence these outcomes indicated (i) technical issues, (ii) the preventive (‘early’) rather than curative (‘late’) potential of anti-amyloid therapies, and (iii) revealed the necessity of multi-target therapies combining anti-amyloid with therapies aiming at Tau – as a “late target” – able to halt the execution of the pathogenetic cascade. Most importantly clinical trials aiming at amyloid plaques highlighted the lack of in depth understanding of (i) the molecular identity of the toxic Aβ (and Tau) forms, (ii) the mechanisms linking both diagnostic pathologies (amyloid and Tau) – and eventually associated pathological features including inflammation, synaptic loss, brain atrophy and (iii) the respective contributions of both pathological proteins to the etiology of AD. Indeed, arising insights encompassed that either not the or not all toxic forms of Aβ were removed, or that administration of the vaccine occurred too late in the disease process, after initiation of a pathogenetic cascade by Aβ, which can be self-propagating and Tau-driven. Alternatively, independent pathogenetic roles of Aβ and Tau were invoked [18]. The interest in Tau as a “late target” for combined therapy – as crucial executor of the degenerative processes – [9] is substantiated by the fact that NFT load and spreading closely correlates with the severity and progression of the disease. Moreover, Tau pathology is associated with a growing list of neurodegenerative diseases referred to as “Tauopathies”. And most importantly, exonic as well as intronic mutations in the Tau gene (MAPT) linked to these Tauopathies [8, 9], demonstrate that Tau is causally linked to neurodegenerative processes. Finally, accumulating evidence indicates that Tau-pathology can spread in a prion-like fashion to different brain regions: the spreading of misfolding of Tau to functionally connected brain regions [19–25], points towards a self-propagating “prion-like” effect – eventually following initiation of the cascade by Aβ. These data position Tau as an important therapeutic target in Tauopathies but also in AD [9], and highlight the need to understand its exact pathological role. Most importantly, the outcome of these trials highlighted the need to understand the relation between Aβ and Tau and their respective contributions to the pathogenetic process.

## Aβ-induced Tau-pathology: experimental support for Aβ-induced Tau-pathology in cellular models, animal models and from patient biomarkers

Despite the many criticisms against the amyloid cascade hypothesis, accumulating evidence obtained in in vitro and in vivo models and in patients provides solid experimental support for the hypothesis, and particularly for Aβ-induced Tau-pathology. More importantly these data position Aβ as accelerator/initiator and Tau as executor of the pathogenetic process, designating their interaction as crucial triggering event in AD. In depth analysis of the mechanisms and the relation between different pathological characteristics and their role in the etiology, should allow the design of fine-tuned therapies for AD with increased efficacy. More particularly, the molecular or physical identity of the toxic form(s) of Aβ (denoted Aβ*), and of Tau (denoted Tau*), temporal and spatial localization of their action(s), (cell-autonomous or not, pre- or post-synaptic, intra- or extra-cellular), the respective contribution of amyloid and Tau to the etiology of AD, and the mechanistic link(s) between amyloid and Tau pathology should be investigated for this purpose.

We here present an overview of in vitro and in vivo models available for further analysis of Aβ-induced Tau-pathology, which remains to be mechanistically resolved unequivocally. The existence of the apparent panoply of different models and their diversity is to be considered a particular asset to delineate those mechanisms that are robustly and consistently linked with Aβ-induced Tau-alterations.

### In vitro models of Aβ-induced Tau alterations

In vitro experiments using various cell types, ranging from neuronal cell lines to primary hippocampal and cortical neurons and hippocampal organotypic cultures, have demonstrated Aβ-induced Tau-alterations. These include increased phosphorylation and cytoplasmic and dendritic translocation, often linked to neurodegeneration which was Tau or even P-Tau dependent [26–38]. Most difficult to identify are the signaling cascades involved and/or the exact identity of Aβ peptides involved. Definition of the exact form of Aβ-peptides is furthermore hampered by the fact that there is a continuous interconversion between different physical forms of the self-assembling Aβ peptides. The variety of forms of amyloid peptides used experimentally, ranged from overexpression of mutant APP, to the extracellular application of oligomeric or fibrillar Aβ_(1–40; 1–42; 1–43, 25–35)_ in concentrations ranging widely. Most experiments were performed with synthetic Aβ [26–30, 32, 33, 35, 37, 38], but also with naturally secreted Aβ or Aβ dimers and oligomers extracted from human AD brains [36]. Application of amyloid peptides increased Tau-phosphorylation and altered Tau conformation to various extent, often associated with or dependent on GSK3 activation [26, 28–31, 35, 38], which was reported to occur downstream of NMDA-receptor signaling [38]. Other signaling pathways have been reported including CAMKK2-AMPK kinase [37] and C-Jun N-terminal kinase [33], further hampering the identification of a common or primary mechanism(s). Finally, neurons differentiated from induced pluripotent stem cells (iPSC) from fibroblasts of 2 patients with familial AD with an APP gene duplication, demonstrated increased Tau phosphorylation at Thr-231, associated with GSK3 activation, while beta-secretase inhibition reduced p-Tau and GSK3 activation [39]. Besides GSK3, several other signaling mechanisms have been implicated in amyloid induced Tau-alterations in primary neurons or other neuronal cell lines, thereby not allowing a conclusive identification of the cascade(s) involved. Although identification and validation of the primary mechanism(s) in in vivo systems is required, compelling evidence – summarized above – indicates that Aβ induces pathologically relevant Tau-alterations in neurons in vitro.

### In vivo models of Aβ-induced Tau-pathology

**Figure 1:**
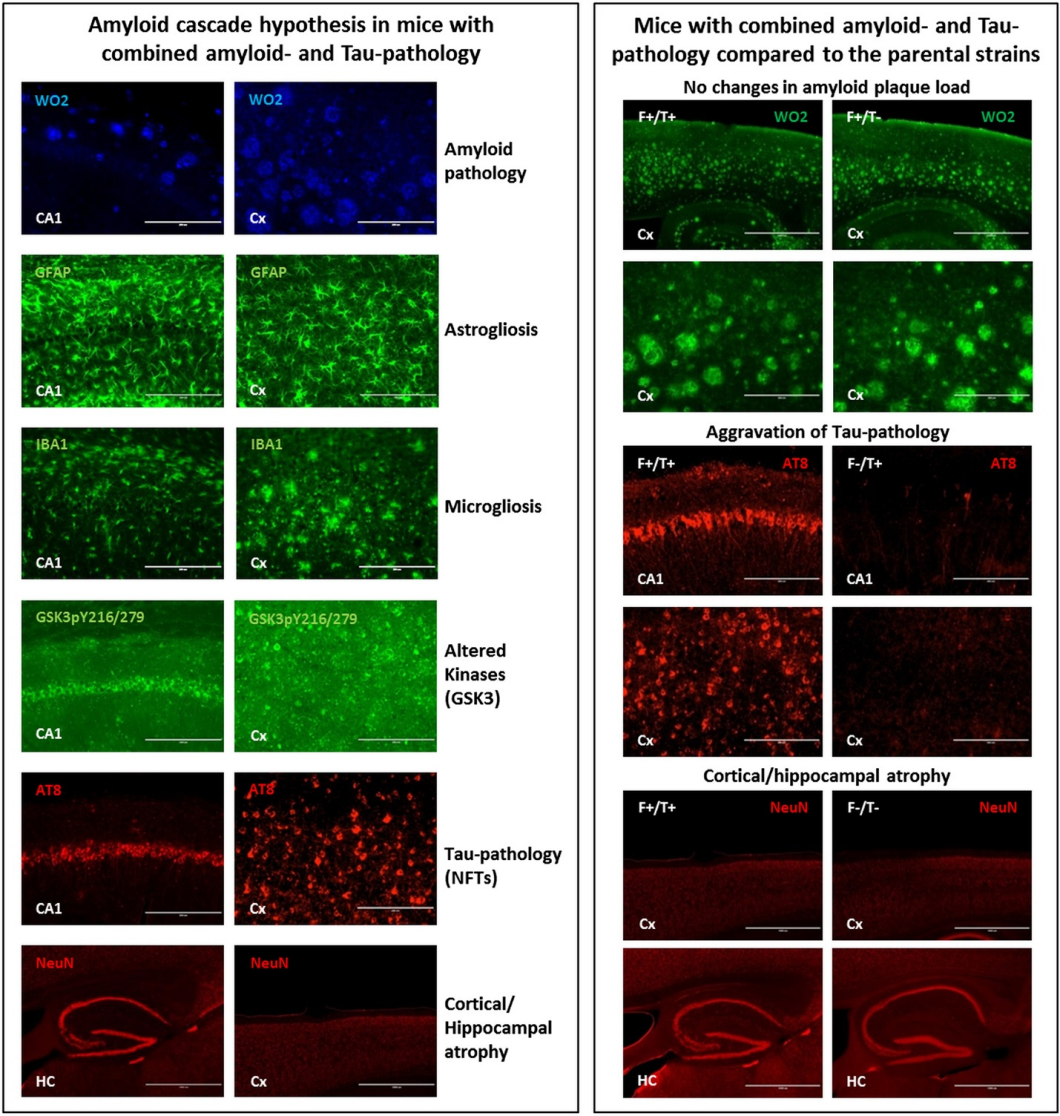
**Amyloid cascade hypothesis in transgenic mice with combined amyloid and Tau pathology (left panel).** Hippocampal (HC; CA1) and cortical (Cx) immunohistochemical staining for amyloid plaques (anti-Aβ, WO2), astrocytes (GFAP), microglia (Iba1), P-GSK3 (GSK3pT216/279), P-Tau (anti-pTau, AT-8) and NeuN for cortical/hippocampal atrophy (all images are 20×, except NeuN at 4×) is presented in transgenic mice expressing mutant APP/PS1 and mutant Tau [55]. These mice were generated by crossing mutant APP/PS1 and mutant Tau mice, respectively denoted as F+/T- (5xFAD; [59]) and F-/T+(TauP301S; [60]). Of note, AT-8 staining was optimized to match Gallyas silver staining patterns, hence representing NFTs (data not shown). Main pathological features found in F+/T+ mice (right panel). Immunolabeling with anti-Aβ (WO2), showing no changes in amyloid plaque load in the cortex of F+/T + and F+/T- mice (upper panel, 4× and 20×); Aggravation of Tau-pathology, anti-pTau (AT-8) staining (NFT), in hippocampal CA1 region and cortex of F+/T+ compared to F-/T+ parental strain transgenic mice (middle panel, 20×); anti-neuronal nuclear staining (NeuN) showing decreased cortical and hippocampal area in F+/T+ compared to F-/T- mice (lower panel, 4×).

**Figure 2:**
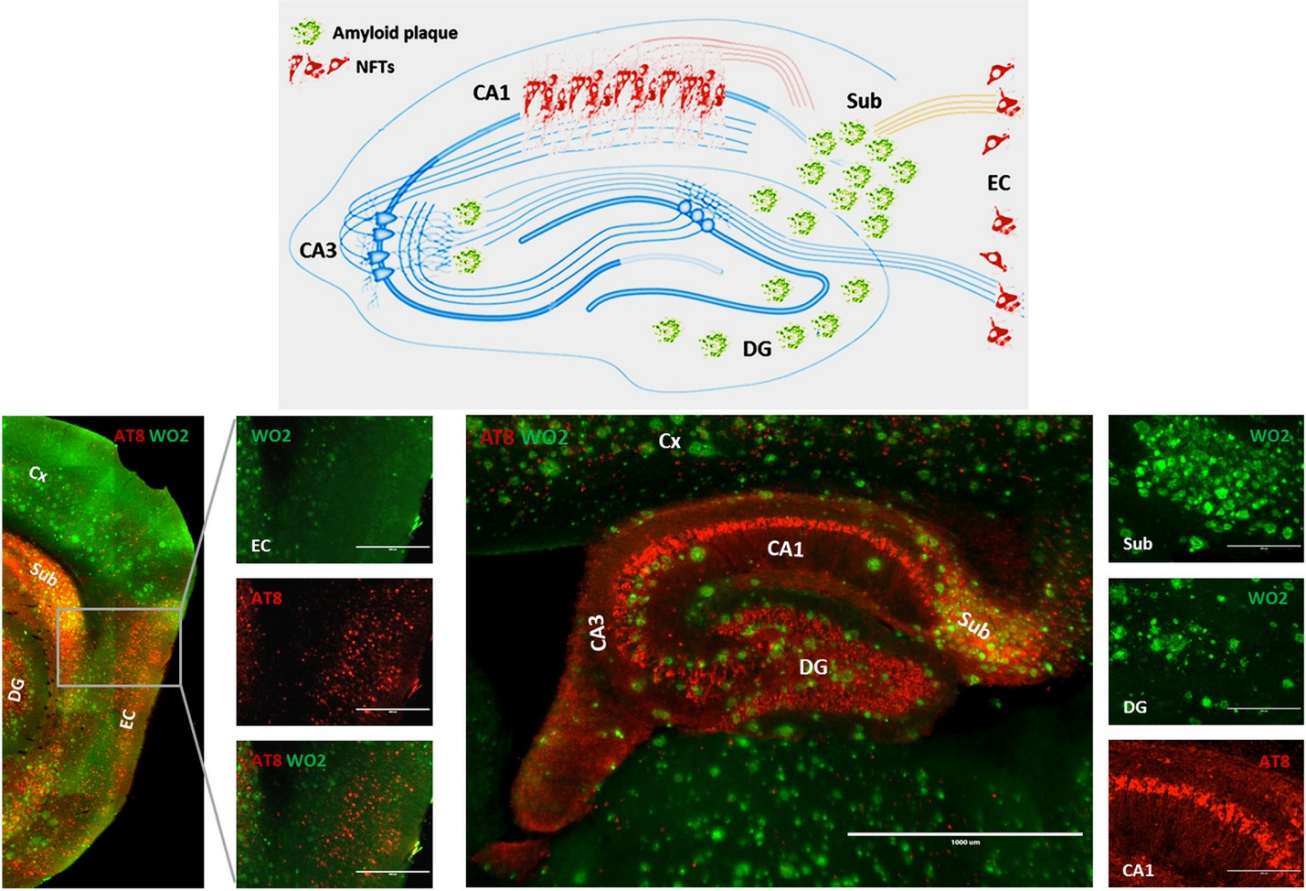
**Graphical representation of the regional distribution of amyloid plaques and NFTs in hippocampus and entorhinal cortex (upper panel, hippocampal-entorhinal connectivity map modified after Deng et al.** [61]**).** Analysis of the relation between amyloid plaques (anti-Aβ, WO2, green) and Tau-pathology (anti-pTau, AT-8, red) in entorhinal cortex (lower left panel) and hippocampus (lower right panel) of F+/T+ transgenic mice, with details of the regional distribution of amyloid- and Tau-pathology for each of these regions.

A growing list of different animal AD models has reproducibly and robustly recapitulated Aβ-induced Tau-pathology. Although initial mouse models expressing mutant APP or mutant APP/PS1 without overexpression of Tau did not display neurofibrillary tangles nor robust Tau aggregation in mouse brain, subtle changes on endogenous mouse Tau induced by high Aβ loads encompassed Tau hyperphosphorylation. Furthermore, models with high plaque loads consistently displayed presence of dystrophic neurites containing hyperphosphorylated, pathological Tau surrounding senile plaques [40–44]. The lack of formation of NFT could be ascribed to the low propensity of endogenous mouse Tau to form NFTs within the life span of mice without Tau overexpression. Transgenic rats overexpressing mutant APP/PS1 displayed increased Tau-alterations in the brains, primarily demonstrated with antibodies against p-Tau and conformationally altered Tau, independent of overexpression of Tau, on the wild-type Tau genetic background [45, 46]. The difference with mouse models might be due to different properties of murine and rat Tau, particularly the expression of different isoforms and/or possibly different levels of expression. Importantly, 3 seminal papers have demonstrated Aβ-induced formation of NFTs in mice overexpressing human mutant Tau [47–49]. Injection of synthetic pre-aggregated Aβ peptides in the brains of Tau transgenic mice, resulted – albeit to a rather limited extent – in the induction of NFTs remote from the injection site, in neurons functionally connected with the site of injection [47]. These data point towards extracellular Aβ as a contributor of amyloid induced Tau-pathology. Secondly, crossing mutant APP transgenic mice with TauP301S mice resulted in increased numbers of NFTs – in female offspring only – [48]. Finally, anti-Aβ immunization in a model with combined amyloid and Tau-pathology reduced early pathological changes in Tau phosphorylation [49, 50]. These findings were further confirmed and extended towards more robust induction of Tau-pathology in different combinations of Tau transgenic mice with transgenic mice overexpressing mutant APP or APP/PS1, and following injection of Aβ peptide enriched brain extracts from mice or patients [51–56]. Furthermore, soluble Aβ and Aβ-oligomers induced Tau-phosphorylation in the brains of wild-type Tau overexpressing mice [57]. More recently, APP/amyloid induced NFT formation was demonstrated in Tau transgenic mice expressing human wild type Tau (3R/4R) [58]. These different results are summarized in Additional file 1: Table S1 and indicate that Aβ-induced Tau-pathology is very reproducible and consistently recapitulated in different models. In contrast, amyloid pathology was either not affected [53–55] or not aggravated by Tau-pathology [56]. The availability of the parental mouse strains thereby allows analysis of the respective contributions to the AD-related phenotypic features. We recently described a model with robust combined amyloid and Tau-pathology, which displayed a dramatic aggravation of Tauopathy compared to the single Tau transgenic mice (Figures 1 and 2). Comparative analysis with the parental amyloid and Tau only mice, demonstrated that aggravated Tau-pathology contributed to synaptic and cognitive deficits and to hippocampal atrophy in this model [55]. Moreover, dramatic hippocampal atrophy was demonstrated and obvious de visu during immunohistochemical analysis, thereby recapitulating an AD pathological feature not displayed in all models in a robust way. Taken together, animal models of AD have reproducibly and robustly recapitulated Aβ-induced Tau-pathology in a variety of different in vivo models, while Tau-pathology did not induce increased amyloid pathology.

### AD patients: an integrated model of biomarker analysis and histopathological analysis: Aβ accelerates antecedent subcortical Tau-pathology

The most relevant data for AD research are undoubtedly patient data, encompassing combined analysis of clinical assessment, postmortem histopathological analysis, genetics and dynamic biomarker analysis. Yet these studies are confronted with more limited possibilities for mechanistic analysis. The obligatory histopathological signature of AD, is the co-occurrence of amyloid plaques and neurofibrillary tangles highlighting the importance of the two respective aggregated proteins Aβ and Tau [1, 4, 5]. Their respective contributions, their chronological importance and interrelation have been a matter of intense animated debates since their discovery (from “Tauists/Baptist” to “chicken or egg”). As elegant discussions and in depth reviews of patient data are provided by others, we restrict the discussion here to a concise description of the integrated hypothesis which reconciles these discussions [1, 62–72]. Dynamic biomarker analysis as indicators of key pathological features in AD, has elegantly addressed the temporal evolution of these processes in relation to each other and to disease progression [65, 71]. Measures of cerebrospinal fluid (CSF) Aβ42 and PET amyloid imaging were used as indicators of brain Aβ deposition. Increased levels of CSF total Tau (t-Tau) and phosphorylated Tau (p-Tau) were taken as indicators of NFT burden in these studies [65, 71]. Hypometabolism on fluorodeoxyglucose (FDG) PET and atrophy on structural MRI as measures of neurodegeneration. Dynamic biomarker analysis revealed a prototypical sequence of biomarker changes in the pathogenic process [65, 71]. Earliest changes appeared in CSF Aβ42, closely followed by amyloid PET imaging with an important lag period before the first symptoms. Alterations in CSF Tau (t-Tau and p-Tau) appear later in the disease process and precede cognitive decline and brain atrophy [65, 71]. Although this sequence is completely in line with the amyloid cascade hypothesis as stated above [1], it was confronted with seemingly contradictory histopathological data demonstrating appearance of Tau-pathology early in life in asymptomatic individuals. Braak et al. demonstrated that Tau-pathology occurs first in limbic regions (entorhinal cortex and CA1) and even earlier in locus coeruleus in brain stem in very young cognitively normal individuals in the absence of amyloid pathology [66]. Progression of Tau-pathology occurs in a stereotypic way, which has been accepted as standard for staging the disease process [5, 73, 74]. This pattern is characterized by first appearance of NFTs in transentorhinal cortex (Stages I-II), subsequent appearance in hippocampal CA1 (II-IV), and subsequent spreading to temporal (V) and isocortical areas (VI) [5, 73, 74]. Although these data demonstrate that Tau-pathology can occur prior to amyloid pathology, neuropathological evaluation by Price and Morris further elaborated these findings. They demonstrated that early Tau-pathology associated with normal ageing, was markedly increased and progressed more rapidly in individuals with Aβ pathology [62, 64, 67, 71]. Aβ pathophysiological changes were thereby proposed “to qualitatively transform and accelerate the antecedent subcortical Tauopathy leading to neocortical spread of NFTs” [62–64, 67, 71].

We would like to integrate in this discussion compelling data from in vitro and in vivo models. Although we take into account the limitations of animal models, often using multiple mutations, overexpression (even of multiple genes sometimes), the invariable demonstration of Aβ-induced Tau-alterations in all these different models [45–57] very strongly support its relevance in the pathogenetic process. Conversely, the fact that in these models Tau-pathology did not affect or aggravate amyloid pathology argues rather against Aβ downstream of Tau as recently proposed [69]. Intriguingly, Aβ accumulation in AD patients occurs temporally and anatomically distinct from Tauopathy and is able to accelerate Tau pathology and to facilitate neurodegeneration [63, 67, 71, 73, 75]. This is strikingly in line with data obtained in animal models [47, 51, 52, 55], demonstrating occurrence of Aβ-induced Tau-pathology along neuronal projections. This includes our model(s), in which NFTs invariably develop early and robust in CA1 region of hippocampus, which is nearly devoid of amyloid plaques but projects to brain regions with high and early plaque load [52, 55] (Figure 2). Similar data, underscoring Aβ-induced Tau-pathology along neuronal connections, were obtained in different models following injection of Aβ [47], or Aβ-containing brain extracts [51] or different APP/Tau mice [52]. In addition, besides a robust aggravation of Tau-pathology also hippocampal atrophy was significantly increased in mice with combined amyloid and Tau-pathology (APP/PS1/Tau mice) compared to the parental strain, with Tau-pathology only [55]. The latter is in line with the fact that in patients amyloid pathology not only accelerates Tau-pathology, but also facilitates associated neurodegeneration [67].

Taken together, data from in vitro and in vivo models and from patient analysis support a model in which Aβ accumulation acts as a triggering event in the pathogenetic process by accelerating antecedent – relatively silent – Tau-pathology. Hence understanding this triggering event of Aβ-induced Tau-pathology is absolutely critical to understand AD and for development of therapeutic strategies, requiring analysis in in vitro and in vivo models.

## In vivo models with Aβ-induced Tau-pathology: tools to explore pathways linking amyloid and Tau-pathology

**Figure 3:**
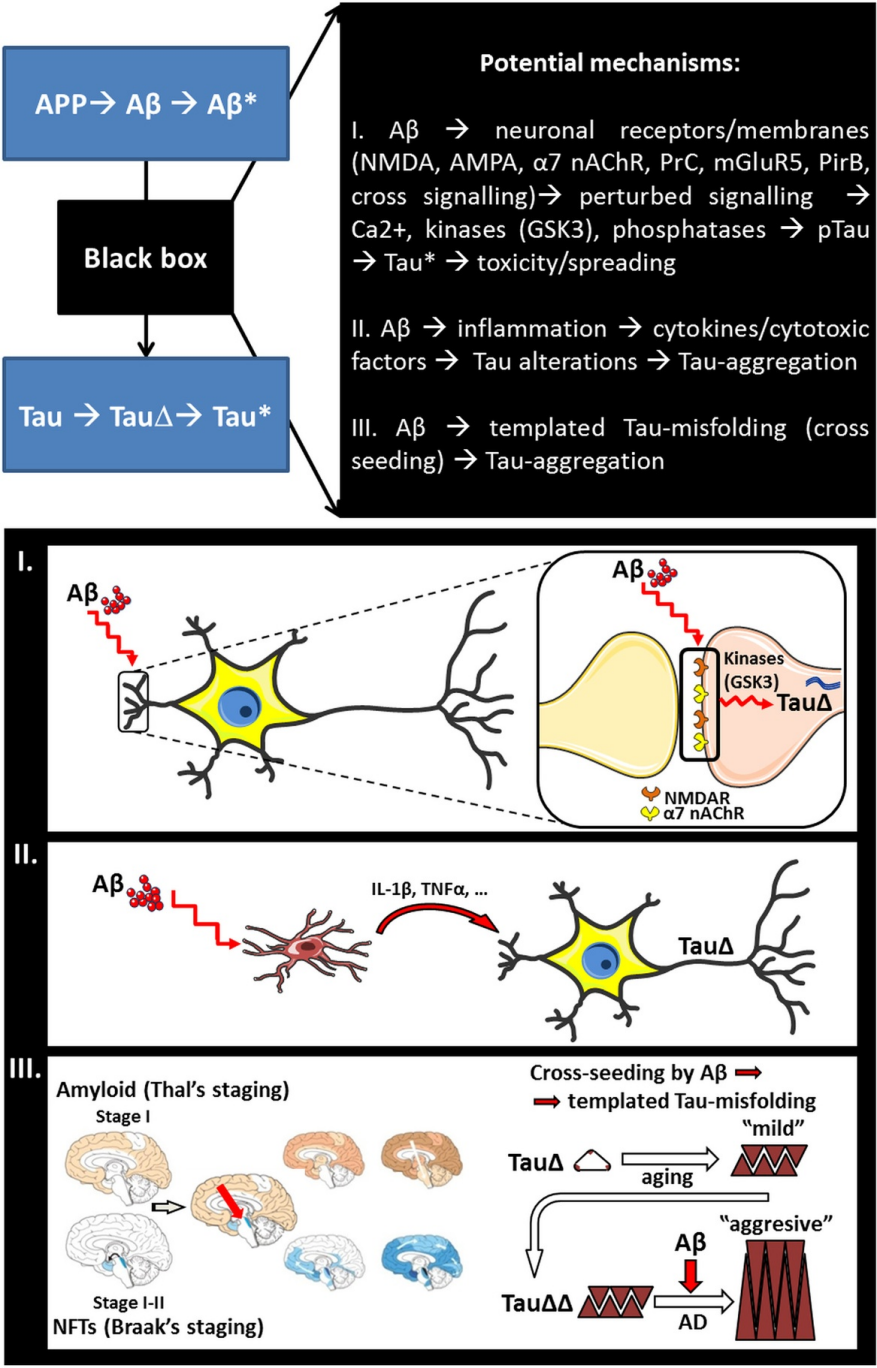
**Schematic presentation of proposed mechanisms involved in Aβ-induced Tau-aggregation as discussed in this review (elements in panel I. and II. modified from Servier Medical Art; elements in panel III. modified from Jucker and Walker** [76] **, Thal et al.** [75] **, Braak and Braak** [73] **).**

Although mechanisms linking amyloid and Tau-pathology have not been conclusively and exhaustively identified, available data support several potential mechanisms that can contribute exclusively or concomitantly. We here use a reductionist approach and limit the discussion to mechanisms which are corroborated or are consistent with experimental data in our models [52, 55] (Figure 3), thereby not excluding contributions of mechanisms not presented here. Theoretically, Aβ peptides may interact directly or indirectly with neurons to induce Tau-alterations. First, direct interactions with neurons that have been reported include specific binding to several neuronal receptors (cfr 4.1) or, because of the sticky nature of amyloid peptides, less specific interaction with membranes and proteins. Secondly, indirect mechanisms may contribute to amyloid induced Tau-pathology, including amyloid induced inflammation via glial cells (cfr 4.2). Finally, in view of the recently observed cross-seeding between misfolded protein species, we need to consider that amyloid peptides may act as direct seeds for Tau-aggregation (cfr 4.3). This latter option has not yet been experimentally explored in detail, although some data are consistent with the hypothesis that pre-aggregated misfolded Aβ peptides could seed and propagate Tau-misfolding and hence aggregation by cross-seeding.

### Direct mechanisms of amyloid induced Tau-alterations – interaction with neuronal receptors and membranes

From the initial definition of amyloid plaques as extracellular Aβ aggregates and NFTs as intraneuronal Tau aggregates, an interaction of extracellular Aβ with neurons through membranes or receptors is proposed to be required for Aβ-induced Tau-pathology. This view, however, was complicated by the identification of intraneuronal forms of Aβ, extracellular forms of Tau and different forms of Aβ peptides as toxic candidate(s). Nevertheless, as injection of amyloid peptides – synthetic or APP mouse brain derived – induce Tau-pathology in Tau transgenic mice [47, 51], extracellularly applied Aβ contributes to Tau alterations hence most straightforward requires interaction with neuronal receptors and/or membranes. Many studies have demonstrated interactions between receptors and Aβ peptides. Binding of Aβ to different types of receptors has been reported, including alpha7 nicotinic acetylcholine receptors (α7 nAChR), NMDA and AMPA receptors – directly or indirectly –, the Ephrin-type B2 receptor (EphB2), insulin receptors, the receptor for advanced glycation end-products (RAGE), the prion protein receptor (PrP-receptor), the mouse paired immunoglobulin-like receptor (PirB) and its human counterpart, leukocyte immunoglobulin-like receptor (LilrB2) [77–90]. In addition, to specific binding of amyloid peptides to receptors, more non-specific binding of the “sticky” amyloid peptides to membranes or membrane-proteins must be considered as a mechanism of action of amyloid toxicity [77].

Monomeric or oligomeric forms of Aβ_1-42_ have been demonstrated to bind with high affinity to α7 nicotinic acetylcholine receptors [78], which was inhibited by α7 nAChR ligands and found to be involved in amyloid induced synaptic and cognitive defects [78, 91]. Depending on the form of the Aβ peptide that binds the receptor, the physiological response varies [78]. In vitro findings using neuroblastoma and ex vivo synaptosomes demonstrated increased Tau-phosphorylation (Ser-202, Thr-181, and Thr-231) following Aβ peptide binding to α7 nAChR, which thereby represents a receptor potentially involved in amyloid induced Tau-pathology. Involvement of α7 nAChR in Aβ-induced Tau-aggregation in animal models remains to be further explored in detail.

Mechanisms involved in Aβ-induced synaptic dysfunction are under meticulous analysis (excellently reviewed in [92–96]). Accumulating evidence supports a role of NMDA-receptors in the etiology of AD, by Aβ mediated effects on synaptic dysfunction and their – indirect – interaction with Aβ peptides [95]. Effects of Aβ on synaptic plasticity resulting in shifting synaptic potentiation (LTP) to synaptic depression (LTD), have been consistently reported in different APP transgenic mice and in different experiments using extracellular application of different forms of – oligomeric – Aβ to hippocampal slices [92–96]. Several studies have indicated downstream effects of amyloid peptides on NMDA-receptor function [95, 97, 98]. Aβ derived diffusible ligands (ADDLs) have been demonstrated to bind to synaptic sites in primary neuronal cultures, co-localizing with but not completely overlapping with NMDA receptors [97, 98]. Hence, Aβ peptides were proposed to indirectly interact with NMDA-receptors, potentially through the EphB2 receptor, a tyrosine kinase receptor known to regulate NMDA receptors or other receptors [88]. Different reports further demonstrated that prolonged Aβ incubation promotes endocytosis of synaptic NMDA receptors – particularly the NR2B subtype –, resulting in depression of NMDA evoked currents and reduced CREB signaling required for long term memory [95, 97–99]. Several mechanisms have been invoked to explain impaired NMDA-function and an “LTP to LTD shift” following Aβ incubation. These included a role for extrasynaptic NR2B receptors, conformational changes of the NMDA receptors, a switch in NMDAR composition from GluN2B to GluN2A [99], changes in downstream signaling cascades or indirectly via mGluR5 [100]. Several studies further indicated a modulatory role of Tau in Aβ-induced excitotoxicity [101] linked to NMDA-receptors. Initially, excitotoxicity, seizures and premature death in APP transgenic mice have been demonstrated to be rescued by Tau-deficiency [102–104]. Follow-up studies further revealed that Tau-dependent targeting of fyn couples the NMDA-receptor to excitoxicity and that mistargetting of fyn mitigates Aβ-toxicity [94, 101, 105–108]. Recent evidence indicates a role for the Aβ -fyn-Tau triad in impaired LTP and cognition [104]. Other toxic effects of Tau and Aβ may be related to this excitotoxity, for instance Aβ-induced defects in axonal transport [109] or mitochondrial dysfunction [110], but may also arise through other yet to be defined mechanisms. Taken together, compelling data support a role of NMDA-receptors in Aβ-induced impaired synaptic plasticity – shifting synaptic potentiation (LTP) to synaptic depression (LTD) pathways.

Of note is the demonstration of a role of GSK3β as regulatory switch between LTD and LTP [111] and the fact that Aβ-induced deficits in LTP are rescued by GSK3β inhibition [112]. Finally, Aβ-induced reduction of NMDA-dependent LTP was linked to increased Tau-phosphorylation and GSK3 activation in hippocampal slices [104]. These data could point to a role of NMDA-receptors in Aβ-induced changes in Tau, possibly via GSK3β. This hypothesis is consistent with – yet not proven by – findings of increased GSK3 activation in 2 different models of Aβ-induced Tau-pathology that we have analyzed [52, 55], and with the fact that inhibition of GSK3 using adeno-associated-viral-mediated knock-down in a model with combined amyloid and Tau-pathology could reduce the latter [113]. Detailed analysis of a potential link between Aβ-induced effects on NMDAR-dependent synaptic dysfunction – or different Aβ-induced synaptic defects – and the amyloid induced pathological alterations in Tau leading to Tau-aggregation needs to be further performed and its consistency and robustness needs to be proven in different models.

Although a complete review of Aβ-receptor or Aβ-membrane interactions is beyond the scope of this work, we here would like to emphasize that unequivocal and consistent identification of the receptor(s) or neuronal membrane interaction and the downstream signaling cascades primarily responsible for amyloid induced Tau-pathology is still lacking. With regard to Aβ-receptors some puzzling questions remain, as previously raised [89, 96] i) Which are the common characteristics of an Aβ receptor? Is there a common structure or sequence that allows its binding? ii) Which are the specific (oligomeric) forms of Aβ that bind to the receptors? iii) Which is the toxic form of Aβ? - Does this form bind to a specific receptor? - and particularly in the context of this review: iv) Does binding of amyloid peptide to this receptor mediate amyloid induced Tau-pathology? and if so v) Via which signaling cascades?

In case of involvement of a neuronal receptor in Aβ-induced Tau-aggregation, the complete pathway between binding of Aβ to this receptor(s) and induced Tau-alterations and Tau-aggregation remains to be identified in detail. Comparative analysis in different models of amyloid induced Tau-pathology to pinpoint this mechanism in an unequivocal way is required and enabled by the availability of different models.

### Amyloid induced inflammation - an inducer of pathological Tau-alterations

Senile plaques are marked by the presence of astro- and microgliosis closely associated with amyloid plaque deposition [3–5]. In addition, astro- and microgliosis are invariably detected in brains of AD patients, originally already described by A. Alzheimer, although not directly used as a diagnostic hallmark for AD [3–5]. In vitro data support a role for micro- and astroglial roles in Aβ-induced Tau-phosphorylation [114–116] as co-cultures with glial cells increased Aβ-induced Tau-phosphorylation in primary neurons [114–116]. Furthermore, in vivo models have invariably and consistently recapitulated astro- and microgliosis induced by amyloid pathology [44, 115]. And, different reports have demonstrated that Tau-pathology is dramatically aggravated by acute and chronic inflammatory insults that induce micro- or astrogliosis [117–119]. It follows that Aβ-induced inflammation can contribute to Aβ-induced Tau pathology. Consistent with this result is the fact that blocking of IL-1 signaling, using an IL-1-R antibody attenuated Tau-pathology in triple transgenic mice [118], while increasing IL-1β exacerbated Tau-pathology [120]. An exhaustive review of the role of inflammation in AD models is beyond the scope of this work, but it will be important to corroborate these findings in different models and particularly to identify the role – and molecular mechanisms – of inflammation in Aβ-induced Tau-pathology.

### Amyloid peptides may act as seeds to qualitatively transform and accelerate antecedent Tau-pathology by “cross-seeding” mechanisms: Transformation of “mild Tau-strains” to “aggressive Tau-strains” by Aβ, triggering accelerated prion-like spreading of Tau-pathology along neuronal circuits – “a working hypothesis”

Recent data have indicated self-propagation of pathogenic protein aggregates in a remarkable variety of neurodegenerative disorders ranging from AD, to Parkinson’s disease, Huntington’s disease to Tauopathies [20, 76, 121]. Specific proteins are misfolded and can subsequently act as seeds that structurally induce misfolding of proteins, causing them to aggregate in pathogenic assemblies ranging from small oligomers to large fibrillar amyloids. In this way, formation of minute amounts of misfolded proteins can act as self-propagating agents which initiate and propagate protein misfolding to functionally connected brain areas [20, 76, 121]. These prion-like spreading properties have recently been demonstrated for Tau [19–22], and could contribute to the characteristic spreading of Tau aggregates, resulting in their spatio- and temporal characteristic spreading to brain regions, as documented by Braak and Braak [73]. Aggregated misfolded Tau extracted from brains of transgenic mice and of different patients suffering from different Tauopathies resulted in seeding and propagation of Tau-pathology in a seed dependent manner in brains of wild type Tau transgenic mice [19, 20, 22]. Similar findings have been reported for misfolded alpha-synuclein in neurons in culture and in mouse brain [122–125]. Intriguingly, misfolded alpha-synuclein seeds were able to initiate and propagate Tau-aggregation, revealing the potential of cross-seeding between different types of misfolded proteins [126]. Several reports have indicated that Aβ peptides also display prion-like seeding [76, 121], enabling initiation and propagation of amyloid plaque formation to remote brain regions. Previous studies demonstrated that Aβ and Tau in vitro can form soluble complexes [127]. Interestingly, in different models, injection of pre-aggregated synthetic amyloid peptides [47], or aggregated amyloid peptide enriched brain extracts [51] induced Tau-aggregation at the injection site but also in functionally connected brain regions remote from the injection site. This indicates that aggregated amyloid peptides can initiate and propagate Tau-aggregation in functionally connected brain regions [47, 51]. In line with these findings, we observed in our model, as described above, a consistent and robust induction of NFT formation (e.g. CA1) in regions with only very scarce plaques, but functionally connected to brain regions with high plaque density (subiculum) [55] (Figure 2). In view of these data, a cross-seeding mechanism of misfolded pre-aggregated Aβ peptides to seed Tau-aggregation should be considered and further experimentally addressed as a potential mechanism of Aβ-induced Tau-pathology. Interestingly, such a mechanism would reconcile histopathological and biomarker data in patients, demonstrating that antecedent Tau-pathology is qualitatively transformed and accelerated by amyloid pathology in anatomically distinct brain regions. Tauopathy thereby becomes qualitatively transformed by cross-seeding by Aβ, accelerating its subsequent spreading along functional brain circuitries. This could be in line with a change of Tau-conformation or “Tau-strain” [25] from a milder (associated with normal ageing) into a more aggressively propagating “Tau-strain” (associated with AD) by cross-seeding or templating by Aβ. The pre-existence of Tau-pathology may thereby be a requirement, underscoring why Aβ pathology specifically affects neurons with pre-existing Tau-pathology (Braak stage I-II), and not nearby neurons. Although this is an appealing hypothesis it needs to be experimentally addressed in detail.

## In vivo models with Aβ-induced Tau-pathology to gain in depth understanding of Aβ-induced Tau-pathology: pathological entities of Aβ and Tau and (cellular) localization

Equally or even more enigmatic than the pathways linking amyloid and Tau remains the identification of the exact molecular and physical (oligomer Aβ^2,3,…x^, fibrils) nature of the toxic forms of amyloid entities involved, as well as whether the mechanism of action is cell-autonomous or non-cell autonomous, intra-neuronal or extracellular, synaptic or extra-synaptic, acting through neuronal connections, or by immediate vicinity. The existence of multiple and different models represents an extra asset to address these questions and confirm a potential mechanism as an important contributor in different models. Models with combined amyloid and Tau pathology and synergism are available and are required to elucidate the mechanisms of Aβ-induced Tau-pathology, through intervention studies, and comparison with the amyloid and Tau pathology only models for deconvolution.

## Conclusion

We here presented accumulating evidence in in vitro models, in vivo models and from biomarkers in patients that supports the amyloid cascade hypothesis, particularly Aβ-induced acceleration of Tau-pathology as a critical trigger in AD. Furthermore, we presented diverse models that recapitulate Aβ-induced Tau-pathology and reviewed some potential contributing mechanisms. This mechanism may be linked to downstream effects of Aβ-induced synaptic defects, or to indirect effects mediated by amyloid induced inflammation. It thereby likely involves interactions of Aβ species with (neuronal) receptors, non-receptor proteins and/or membranes, that need to be identified. Furthermore, cross-seeding of misfolded proteins, in which Aβ cross-seeding of Tau, induces transition from mild Tau-strains to more aggressive Tau-strains and thereby triggers prion-like spreading of Tauopathy along neuronal circuitries, was suggested as a potential mechanism. This was based on the distinct spatio-temporal distribution of amyloid and Tau-pathology, which was also observed in transgenic AD models. A unifying mechanism of amyloid induced Tau-pathology still needs to be identified, which reconciles different previous data-sets, and which can be consistently and unequivocally demonstrated in different models with amyloid induced Tau-pathology. Most importantly, in depth understanding of Aβ-induced Tau-pathology in terms of identification of the exact molecular entity, exact molecular mechanism, and their respective contributions to and interrelation with associated pathological features (synaptic dysfunction, neurodegeneration, brain atrophy, inflammation) is absolutely required to define fine-tuned therapeutic strategies with a higher success in preventing or halting AD.

## Electronic supplementary material

Additional file 1: Table S1: Aβ induced Tau pathology in in vivo models*. (PDF 362 KB)
