# Supplementary material for: Models of β-amyloid induced Tau-pathology: the long and “folded” road to understand the mechanism
Source: Mol Neurodegener. 2014 Nov 18;9:51. doi: 10.1186/1750-1326-9-51 (PMC4255655; doi:10.1186/1750-1326-9-51)
Supplement: Supplementary file 1 — Additional file 1: Table S1: Aβ induced Tau pathology in in vivo models*. (PDF 362 KB) [file 13024_2014_562_MOESM1_ESM.pdf]

Table S1

Aβ induced Tau pathology in in vivo models*

Model	Biological Relevance	References
**Transgenic mice with amyloid pathology only**<br>(a panoply of models)<br>(APP^{EOFAD}, $APP^{EOFAD}xPS1^{EOFAD}$)	Models with high plaque load display dystrophic neurites containing hyperphosphorylated Tau surrounding amyloid plaques ("senile plaques"). Some models display hyperphosphorylated Tau. None of the models developed full blown NFTs	(Hsiao, 1998, Duyckaerts et al., 2008)<br>[1, 2]<br>(**refs)
**Double transgenic mouse model**<br>(Tau^{P301L} x $APP^{KM670/671NL}$)	Aggravated Tau-pathology (NFTs) in double transgenic mice compared to the parental Tau strain	(Lewis et al., 2001)<br>[3]
**Tau^{P301L} transgenic mice injected with Aβ42 fibrils**<br>(Tau^{P301L} + synthetic Aβ42 fibrils)	NFTs develop in neurons projecting to the site of injection of synthetic Aβ42 fibrils in TauP301L mice.	(Gotz et al., 2001)<br>[4]
**Triple transgenic mouse model**<br>($APP^{KM670/671NL}$, $PS1^{M146V}$ and Tau^{P301L})	Amyloid pathology preceded tau pathology<br><br>Aβ immunotherapy reducing extracellular and intracellular Aβ, leads to reduced early tau pathology	(Oddo et al., 2003, Oddo et al., 2004)<br>[5, 6]
**Transgenic rat model**<br>($APP^{KM670/671NL}$ and APP^{V717F})<br>($APP^{KM670/671NL}$ and $PS1^{M146V}$)	Tau-alterations in APP transgenic rats, without overexpression of Tau	(Echeverria et al., 2004)<br>[7]
**Tau^{P301L} mice injected with brain lysate of $APP^{KM670/671NL}$**<br><br>**Double transgenic mouse model**<br>(Tau^{P301L} x $APP^{KM670/671NL}$)	Induction of Tau-pathology (NFTs) in Tau mice injected with Aβ-containing brain extracts of APP transgenic mice. NFT appear at the site of injection and in remote brain regions.<br>Aggravated tau pathology (NFT) in the double transgenic compared with the parental Tau mice	(Bolmont et al., 2007)<br>[8]
**Double transgenic mouse model**<br>(APP^{V717I} x Tau^{P301L})<br><br>**Double transgenic mouse model**<br>(GSK-3β x Tau^{P301L})	Aggravated Tau-pathology (NFTs) in hippocampus and cortex in double transgenic (APP x Tau) mice compared to the parental Tau strain.<br>Overexpression of constitutive active GSK3β, causes aggravation of Tau-pathology (NFTs) in hippocampus and cortex	(Terwel et al., 2008)<br>[9]
**Double transgenic mouse model**<br>(Tau^{P301S} x APP^{V717F})	Robustly aggravated Tau-pathology (NFTs) in double transgenic mice compared to parental Tau mice. Development of NFTs followed a spatiotemporal Braak-like pattern	(Hurtado et al., 2010)<br>[10]

**Double transgenic mouse model** (APP^{E693G} x Tau WT)	Reduction of Aβ (BACE inhibition) rescued cognition and reduced somatic accumulation and phosphorylation wild type Tau.	(Chabrier et al., 2012) [11]
**Transgenic rat model** ($APP^{KM670/671NL}$ and $PS1^{\Delta E9}$ genes)	Pathological Tau-changes in rats (endogenous Tau), overexpressing mutant APP/PS1	(Cohen et al., 2013) [12]
**Double transgenic mouse model** ($APP^{KM670/671NL;\ I716V;\ V717I}$, $PS1^{M146L;\ L286V}$ x Tau^{P301S})	Robustly aggravated Tau-pathology (NFT) and neuronal loss in double transgenic mice compared to parental Tau strain	(Saul et al., 2013) [13]
**Double transgenic mouse model** ($APP^{KM670/671NL;\ I716V;\ V717I}$, $PS1^{M146L;\ L286V}$ x $Tau^{G272V/P301S}$)	Robustly aggravated NFT pathology in double transgenic mice compared to parental Tau strain, associated with increased phosphorylation and truncation of Tau	(Heraud et al., 2014) [14]
**Double transgenic mouse model** ($APP^{KM670/671NL;\ I716V;\ V717I}$, $PS1^{M146L;\ L286V}$ x Tau^{P301S})	Robustly aggravated Tau-pathology, and increased cognitive deficits, synaptic deficits and hippocampal atrophy in the double transgenic mice compared with parental Tau strain	(Stancu et al., 2014) [15]
**Double transgenic mouse model** ($APP^{E693\Delta}$ x Tau WT)	Aggravation of Tau-pathology (NFT) in double transgenic mice compared to the parental Tau strain, mildly overexpressing human wild type Tau (3R/4R)	(Umeda et al., 2014) [16]

*We do not claim to provide an exhaustive review of all existing models in this table. We here summarize accumulating evidence in different models demonstrating that Aβ induces Tau-alterations, including pathologically relevant Tau-hyperphosphorylation, somatodendritic accumulation of Tau and full-blown NFT formation. This is not only demonstrated with overexpression of mutant Tau, but also with wild type human Tau, and without Tau overexpression. Furthermore, although some models are based on expression of mutant APP or APP/PS1, injection of extracellular Aβ was sufficient to induce Aβ-induced NFTs. Taken together, these data support Aβ-induced Tau-pathology as a reproducible event in AD models, which must be taken in consideration.

**Multiple APP and APP/PS1 transgenic mice have been generated and characterized in detail (PDAPP, Tg2576, APP23, C3-3 line, CRND8, hAPPH6, J9 hAPP, APP/Ld, J20 hAPP, APP dutch line, ARC6 and ARC48) [9, 17-26]. None of these models displayed full blown NFTs, but dystrophic neurites containing hyperphosphorylated Tau surrounding senile plaques were detected in models with sufficiently high plaque load ("senile plaques"). Some models displayed mild increased levels of hyperphosphorylated Tau [19, 26].

References

1. Hsiao K: **Transgenic mice expressing Alzheimer amyloid precursor proteins**. *Experimental gerontology* 1998, **33**(7-8):883-889.
2. Duyckaerts C, Potier MC, Delatour B: **Alzheimer disease models and human neuropathology: similarities and differences**. *Acta neuropathologica* 2008, **115**(1):5-38.
3. Lewis J, Dickson DW, Lin WL, Chisholm L, Corral A, Jones G, Yen SH, Sahara N, Skipper L, Yager D *et al*: **Enhanced neurofibrillary degeneration in transgenic mice expressing mutant tau and APP**. *Science* 2001, **293**(5534):1487-1491.
4. Gotz J, Chen F, van Dorpe J, Nitsch RM: **Formation of neurofibrillary tangles in P301l tau transgenic mice induced by Abeta 42 fibrils**. *Science* 2001, **293**(5534):1491-1495.
5. Oddo S, Caccamo A, Kitazawa M, Tseng BP, LaFerla FM: **Amyloid deposition precedes tangle formation in a triple transgenic model of Alzheimer's disease**. *Neurobiology of aging* 2003, **24**(8):1063-1070.
6. Oddo S, Billings L, Kesslak JP, Cribbs DH, LaFerla FM: **Abeta immunotherapy leads to clearance of early, but not late, hyperphosphorylated tau aggregates via the proteasome**. *Neuron* 2004, **43**(3):321-332.
7. Echeverria V, Ducatenzeiler A, Dowd E, Janne J, Grant SM, Szyf M, Wandosell F, Avila J, Grimm H, Dunnett SB *et al*: **Altered mitogen-activated protein kinase signaling, tau hyperphosphorylation and mild spatial learning dysfunction in transgenic rats expressing the beta-amyloid peptide intracellularly in hippocampal and cortical neurons**. *Neuroscience* 2004, **129**(3):583-592.
8. Bolmont T, Clavaguera F, Meyer-Luehmann M, Herzig MC, Radde R, Staufenbiel M, Lewis J, Hutton M, Tolnay M, Jucker M: **Induction of tau pathology by intracerebral infusion of amyloid-beta -containing brain extract and by amyloid-beta deposition in APP x Tau transgenic mice**. *The American journal of pathology* 2007, **171**(6):2012-2020.
9. Terwel D, Muyllaert D, Dewachter I, Borghgraef P, Croes S, Devijver H, Van Leuven F: **Amyloid activates GSK-3beta to aggravate neuronal tauopathy in bigenic mice**. *The American journal of pathology* 2008, **172**(3):786-798.

10. Hurtado DE, Molina-Porcel L, Iba M, Aboagye AK, Paul SM, Trojanowski JQ, Lee VM: **A{beta} accelerates the spatiotemporal progression of tau pathology and augments tau amyloidosis in an Alzheimer mouse model**. *The American journal of pathology* 2010, **177**(4):1977-1988.

11. Chabrier MA, Blurton-Jones M, Agazaryan AA, Nerhus JL, Martinez-Coria H, LaFerla FM: **Soluble abeta promotes wild-type tau pathology in vivo**. *The Journal of neuroscience : the official journal of the Society for Neuroscience* 2012, **32**(48):17345-17350.

12. Cohen RM, Rezai-Zadeh K, Weitz TM, Rentsendorj A, Gate D, Spivak I, Bholat Y, Vasilevko V, Glabe CG, Breunig JJ *et al*: **A transgenic Alzheimer rat with plaques, tau pathology, behavioral impairment, oligomeric abeta, and frank neuronal loss**. *The Journal of neuroscience : the official journal of the Society for Neuroscience* 2013, **33**(15):6245-6256.

13. Saul A, Sprenger F, Bayer TA, Wirths O: **Accelerated tau pathology with synaptic and neuronal loss in a novel triple transgenic mouse model of Alzheimer's disease**. *Neurobiology of aging* 2013, **34**(11):2564-2573.

14. Heraud C, Goufak D, Ando K, Leroy K, Suain V, Yilmaz Z, De Decker R, Authelet M, Laporte V, Octave JN *et al*: **Increased misfolding and truncation of tau in APP/PS1/tau transgenic mice compared to mutant tau mice**. *Neurobiology of disease* 2014, **62**:100-112.

15. Stancu IC, Ris L, Vasconcelos B, Marinangeli C, Goeminne L, Laporte V, Haylani LE, Couturier J, Schakman O, Gailly P *et al*: **Tauopathy contributes to synaptic and cognitive deficits in a murine model for Alzheimer's disease**. *FASEB journal : official publication of the Federation of American Societies for Experimental Biology* 2014, **28**(6):2620-2631.

16. Umeda T, Maekawa S, Kimura T, Takashima A, Tomiyama T, Mori H: **Neurofibrillary tangle formation by introducing wild-type human tau into APP transgenic mice**. *Acta neuropathologica* 2014, **127**(5):685-698.

17. Games D, Adams D, Alessandrini R, Barbour R, Berthelette P, Blackwell C, Carr T, Clemens J, Donaldson T, Gillespie F *et al*: **Alzheimer-type neuropathology in transgenic mice overexpressing V717F beta-amyloid precursor protein**. *Nature* 1995, **373**(6514):523-527.

18. Hsiao K, Chapman P, Nilsen S, Eckman C, Harigaya Y, Younkin S, Yang F, Cole G: **Correlative memory deficits, Abeta elevation, and amyloid plaques in transgenic mice**. *Science* 1996, **274**(5284):99-102.

19. Sturchler-Pierrat C, Abramowski D, Duke M, Wiederhold KH, Mistl C, Rothacher S, Ledermann B, Burki K, Frey P, Paganetti PA *et al*: **Two amyloid precursor protein transgenic mouse models with Alzheimer disease-like pathology**. *Proceedings of the National Academy of Sciences of the United States of America* 1997, **94**(24):13287-13292.

20. Borchelt DR, Thinakaran G, Eckman CB, Lee MK, Davenport F, Ratovitsky T, Prada CM, Kim G, Seekins S, Yager D *et al*: **Familial Alzheimer's disease-linked presenilin 1 variants elevate Abeta1-42/1-40 ratio in vitro and in vivo**. *Neuron* 1996, **17**(5):1005-1013.

21. Borchelt DR, Ratovitski T, van Lare J, Lee MK, Gonzales V, Jenkins NA, Copeland NG, Price DL, Sisodia SS: **Accelerated amyloid deposition in the brains of transgenic mice coexpressing mutant presenilin 1 and amyloid precursor proteins**. *Neuron* 1997, **19**(4):939-945.

22. Chishti MA, Yang DS, Janus C, Phinney AL, Horne P, Pearson J, Strome R, Zuker N, Loukides J, French J *et al*: **Early-onset amyloid deposition and cognitive deficits in transgenic mice expressing a double mutant form of amyloid precursor protein 695**. *The Journal of biological chemistry* 2001, **276**(24):21562-21570.

23. Mucke L, Masliah E, Yu GQ, Mallory M, Rockenstein EM, Tatsuno G, Hu K, Kholodenko D, Johnson-Wood K, McConlogue L: **High-level neuronal expression of abeta 1-42 in wild-type human amyloid protein precursor transgenic mice: synaptotoxicity without plaque formation**. *The Journal of neuroscience : the official journal of the Society for Neuroscience* 2000, **20**(11):4050-4058.

24. Herzig MC, Winkler DT, Burgermeister P, Pfeifer M, Kohler E, Schmidt SD, Danner S, Abramowski D, Sturchler-Pierrat C, Burki K *et al*: **Abeta is targeted to the vasculature in a mouse model of hereditary cerebral hemorrhage with amyloidosis**. *Nature neuroscience* 2004, **7**(9):954-960.

25. Cheng IH, Palop JJ, Esposito LA, Bien-Ly N, Yan F, Mucke L: **Aggressive amyloidosis in mice expressing human amyloid peptides with the Arctic mutation**. *Nature medicine* 2004, **10**(11):1190-1192.

26. Moechars D, Dewachter I, Lorent K, Reverse D, Baekelandt V, Naidu A, Tesseur I, Spittaels K, Haute CV, Checler F *et al*: **Early phenotypic changes in transgenic mice that overexpress different mutants of amyloid precursor protein in brain**. *The Journal of biological chemistry* 1999, **274**(10):6483-6492.
